# Nip the HPV encoded evil in the cancer bud: HPV reshapes TRAILs and signaling landscapes

**DOI:** 10.1186/1475-2867-13-61

**Published:** 2013-06-17

**Authors:** Talha Abdul Halim, Ammad Ahmad Farooqi, Farrukh Zaman

**Affiliations:** 1 Department of Obstetrics & Gynaecology, RLMC, 35 Km Ferozepur Road, Lahore, Pakistan; 2 Laboratory for Translational oncology and Personalized Medicine, RLMC, 35 Km Ferozepur Road, Lahore, Pakistan

## Abstract

HPV encoded proteins can elicit ectopic protein–protein interactions that re-wire signaling pathways, in a mode that promotes malignancy. Moreover, accumulating data related to HPV is now providing compelling substantiation of a central role played by HPV in escaping immunosurveillance and impairment of apoptotic response. What emerges is an intricate network of Wnt, TGF, Notch signaling cascades that forms higher-order ligand–receptor complexes routing downstream signaling in HPV infected cells. These HPV infected cells are regulated both extracellularly by ligand receptor axis and intracellularly by HPV encoded proteins and impair TRAIL mediated apoptosis. We divide this review into different sections addressing how linear signaling pathways integrate to facilitate carcinogenesis and compounds that directly or indirectly reverse these aberrant interactions offer new possibilities for therapy in cancer. Although HPV encoded proteins mediated misrepresentation of pathways is difficult to target, improved drug-discovery platforms and new technologies have facilitated the discovery of agents that can target dysregulated pathways in HPV infected cervical cancer cells, thus setting the stage for preclinical models and clinical trials.

## Introduction

There is an overwhelming list of research work that underlines the fact that HPV encoded proteins control cell cycle progression, apoptosis and cell differentiation, and have emerged as fundamental regulators of cervical cancer. Recent studies have revealed a complex network of protein interactions in HPV infected cells, and have connected HPV encoded proteins with other key signaling pathways. Such crosstalk has uncovered novel roles for signalings, including regulation of TGFβ/SMAD, WNT/β-catenin and Notch signaling cascades by HPV encoded proteins during carcinogenesis. This review highlights recent findings and trends in the HPV infected cervical cancer with an emphasis on how the HPV encoded proteins integrate with other pathways to promote cervical cancer. Moreover, several clues related to role of TRAIL mediated signaling in HPV infected cervical cancer cells are discussed. It also provides a better understanding of role of miRNAs in HPV infected cervical cancer cells. We also review recent patterns and approaches which have been used to induce apoptosis in HPV infected cervical cancer cells. Oncogenic proteins, such as those encoded by HPV, frequently form ectopic signaling complexes to re-constitute cellular behavior and exemplify how improved understanding of the HPV associated mechanisms might be translated into clinical benefit.

Histological studies provide classification of cervical cancer. Therefore it can be characterized into different sub-categories, including squamous cell carcinomas (SCC) and adenocarcinomas (AdCAs). SCCs develop via well-defined precursor stages, called cervical intraepithelial neoplasia (CIN, graded 1–3), however precursor stages for AdCAs are less well characterized. The precursor lesions to cervical cancers are known as cervical intraepithelial neoplasia (CIN) and noatably CIN1 lesions are referred to as low-grade CIN whereas CIN2 and 3 lesions together are considered high-grade CIN [1,2].

Epidemiological and experimental studies have provided considerable verification that persistent infections with high-risk types of HPV (hrHPVs; HPV16, 18, 31, 33, 45) are causative agents of cervical cancer [3]. Increasing sophisticated information has enhanced our knowledge related to HPV-16 genome. It is a well established fact that HPV-16 genome is organized into six early (E1, E2, E4, E5, E6, and E7) and two late (L1 and L2) open reading frames that code for functional and structural proteins, respectively. There is a categorization based on functions of the proteins as E1 and E2 are necessary for replication of the viral genome, E6 and E7 are responsible for maintaining the correct environment for DNA replication in the host cell by preventing possible cell cycle arrest and intrinsic p53-dependent apoptosis [4]–[6].

**Figure 1:**
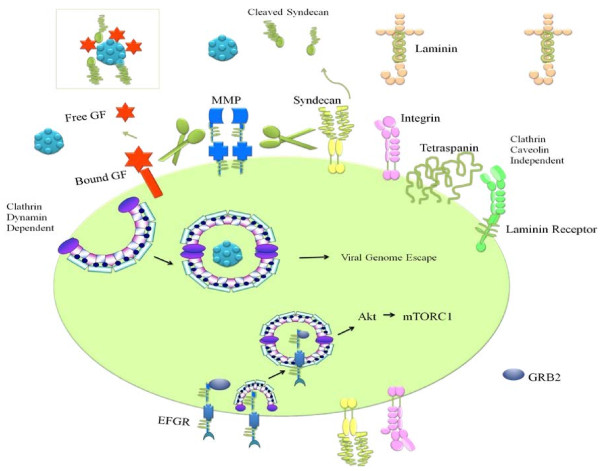
**Heparan sulfate proteoglycans (HSPGs) are primary attachment molecules for entry of Human Papilloma Viruses into cells.** Syndecans are cell surface heparan sulfates. Laminin undergoes proteolytic processing and can interact with different cell surface receptors including integrin, syndecan and laminin receptor. CD151 is a member of the tetraspanin group of transmembrane proteins and can interact with different integrin α subunits, including α3, α6, and α7. EGFRs are internalized and there is a prolonged receptor mediated signaling in HPV infected cells. Matrix Metalloproteinases proteolytically cleave bound growth factors and syndecans. These cleaved moieties bind to HPV and this complex can interact with different receptors to gain entry into the cervical cells.

Accumulating evidence on cellular receptor-binding and internalization pathways of HPVs is providing further insights into the function of the pathways involved, their constituent proteins and ways in which they gain entry into host cells. There are wide-ranging pathways which are documented to be used by HPV including clathrin-mediated endocytosis, caveolar endocytosis, clathrin- and caveolae-independent pathway. Details can be found elsewhere [7,8]. It has previously been speculated that virus enters into host cell simplistically however it is now evident that HPV interacts with different molecules extracellularly for rapid activation of signaling pathways important for infection. It has been experimentally verified that HPV interaction with syndecan-1 via HSPG and binding of syndecan-1 to laminin 332 and α6β4 integrin are in accordance with the notion that HPV particles colocalize and interact with each of these extracellular molecules [9]. Similarly, CD151-associated integrins (α3β1 and α6β1/4) also regulate HPV16 infection [10]. HPV16 E6 activated mTORC1 by enhanced signaling through miscellaneous receptors, including EGFR, Insulin Receptor and insulin-like growth factor receptors. It was shown that there was a prolonged internalized receptor and a gradual decline in cell surface appearance of those receptors [11]. It has been reported that HPV16 infection is lowest in α6 integrin null cells and experimental methodologies have revealed that Focal Adhesion Kinase (FAK) is the protein activated upon integrin binding. HPV16 induces FAK-Tyr397 phosphorylation in cancer cells and it is intriguing to note that targeted inhibition of α6 integrin function prevents FAK-Tyr397 phosphorylation [12]. In line with the same concept it is appropriate to mention that α2β1 integrin promotes tumor metastasis in HPV-induced squamous cancer, probabilistically by promoting migratory and invasive potential of cells [13]. However molecular mechanisms are not studied in detail in cervical cancer cells. Overview of HPV entry into cervical cells is shown in the Figure 1.

Studies of cervical neoplasia suggest that HPV infection alone is not responsible for tumor development rather Common Fragile Sites (CFSs) are preferential targets for HPV integration in cervical tumors. Convincing substantiation of this relationship was first provided when the sequence of the FRA3B region at 3p14.2 and cellular sequences flanking an HPV16 integration in a cervical tumor were indicated to be identical [14]. It has also been documented that HPV16 integration in CFS results in loss of tumor suppressor genes [15]. E7 oncoprotein interacts primarily with the pATM and it is appropriate to mention that ATM pathway is utilized by HPVs to promote viral replication in differentiating cells [16]. However there is a conflicting report that suggested that ATM mediated signaling induced cell cycle arrest in cervical cancer cells. Furthermore, cervical cancer cells treated with isoliquiritigenin (ISL) displayed an activated ATM that activated its downstream effectors. In addition there was considerably enhanced ratio of pro-apoptotic proteins Hsu et al, [17].

Sp1 DNA binding sites are present within HPV promoters and play an active role in HPV gene expression. Cells expressing HPV promoter luciferase reporter vector treated with nordihydroguaiaretic acid plant lignan derivatives displayed remarkable repression of HPV encoded genes [18].

It is of particular significance that HPV 16 E5 oncogene is a small, highly hydrophobic protein of 83 amino-acids that localizes in endocellular membrane and undergoes homo-oligomerization. This process of oligomerization creates a hydrophilic pore that allows passage of small molecules and ions through these channels [19]. It has been experimentally verified that HPV16 E5 interferes with degradative and recycling endocytic pathways of receptors. E5 protects degradation of receptors by inhibiting the recruitment of Grb2-Cbl complexes responsible for receptor ubiquitination and degradation [20]. It is persuasive to note that E5 regulates the expression of IFN-β via IRF-1 and knockdown of IRF-1 expression in E5-expressing cells abolishes IFN-β expression. Astonishingly, stimulation of IRF-1 expression by HPV16 E5 occurs through E5 mediated activation of NF- κB that moves into the nucleus to attach to consensus sequences on the IRF-1 gene promoter [21].

Autophagy or "self-eating" is an important mechanism and it has been shown that cellular decisions to autophagy manipulation are modulated by HPV. Cells infected with HPV16 displayed activated PI3K/Akt/mTOR pathway that inhibited autophagy [22]. RECK is a membrane bound protein and is a negative regulator of MMPs. However it has been shown that E6 and E7 down-regulate RECK and promote activity of MMP9. One possible mechanism could be miRNA mediated control of RECK in cervical cancer cells [23]. Structural studies provide reasonable evidence that conserved regions of E7 are involved in interaction with different proteins. Transcriptional co-activator p300, which contains an intrinsic HAT activity, is essential for wide ranging biological functionalities is reported to be regulated by E7 via its CR1 and CR2 domains [24]. Similarly, E7 interacts with pRb through its CR2 and CR3 domains [25]. TBX2 and TBX3 are members of the T-box family of transcription factors and have been reported to repress transcription from the LCR via interaction with HPV16 L2. CHIP analysis provided strong evidence of co-localization of L2 and TBX2 in HPV16 positive CIN I-II tissue sections [26].

ChIP assay confirm that HPV-18 E2 binds the hTERT promoter region via Sp1in vivo and represses the expression of hTERT. However, HPV-16 E2 stimulates the hTERT expression [27,28]. E5 is interconnected with downregulation of antigen presentation by HLA class I molecules, a protective mechanism that promotes HPVs ability to evade immune clearance through cytotoxic-T-lymphocyte (CTL)-mediated adaptive immunity. E5 restricts HLA-A and -B molecules in the golgi apparatus thus repressing their cell surface appearance [29]. Similar mechanisms are opted by HPV to regulate CD1d, an (MHC) class I-like glycoprotein. Cellular studies suggest that E5-expressing epithelial cells retain CD1d in the ER via interfering with the modification of HLA class I heavy chains that characteristically takes place in the ER. E5 has been documented to interact with calnexin in the ER thus compromising calnexin-mediated CD1d folding and impairing trafficking of CD1d to the surface of HPV-infected cells [30].

HPV encoded E5 protein utilizes cAMP/PKA/CREB pathway to stimulate the expression of Prostaglandin E2 (PGE2) receptor [31]. One of the best-characterized biological effects of 16E5 is ERK activation and it has additionally shown that ERK specific phosphorylation sites are present in E1-E4 protein. Predominantly, phosphorylation at threonine 57 enhances keratin binding and provides protection against proteasomal degradation [32]. It has previously been reported that HPV 16E1-E4 protein induces G2 arrest. It was further underscored that arrest does not result from inhibition of the kinase activity of the Cdk1/cyclin B1 complex rather due to retention of active Cdk1/cyclin B1 complexes in the cytoplasm away from their nuclear substrates [33].

Spindle assembly checkpoint (SAC) is a 'wait-anaphase' mechanism that has evolved in eukaryotic cells and spindle checkpoint proteins (SCPs), sense the existence of misaligned sister chromatids during mitosis and meiosis. HPV16/18 E5 expressing cells have considerably reduced expression of Bub1 and Mad2 [34]. NIH 3T3 cells transfected with the HPV 16 full-length genome and mimetic miR-125b displayed drastic decrease in viral DNA and protein synthesis however, co-transfection with anti-miR-125b and HPV 16 markedly increased HPV DNA [35]. miR-125b also promotes cell death by negatively regulates spindle assembly checkpoint gene MAD1 [36].

Various splicing factors including ASF/SF2 are over-expressed in high-grade cervical lesions and cervical cancer. It was indicated that E2 caused a three- to fourfold upregulation of SF2/ASF [37]. Using experimental techniques it has been identified that 3'-splice site on the HPV-16 genome, is used to produce primarily E4, E6, and E7 mRNAs and is regulated by ASF/SF2. More specifically, splice site is followed by 15 potential binding sites for the splicing factor ASF/SF2 [38]. Rapidly increasing research on post-transcriptional regulation of HPV 16 indicates that hnRNPA1 and hnRNPA2 promote HPV16 E6 exon exclusion, whereas Brm and Sam68 mediate exon inclusion [39]. It has also been suggested that hnRNP A1 binding to the HPV-16 late 3′ splice site prevents the interaction of the splice site with the U2AF35/U2AF65 factors, thus inhibiting splicing [40].

Laboratory analysis of immortal human cell lines transfected with E6 also suggests that E6 oncoproteins are characterized by the presence of a PDZ (PSD95/Dlg/ZO-1) binding motif in their extreme carboxy termini. PDZ domain-containing cellular substrates, including the cell polarity regulators human Dlg (hDlg) (11) and human Scribble (hScrib) have been identified to be known targets of E6. Furthermore, other E6 PDZ domain-containing targets consist of the MAGI family of proteins, which act as scaffolds in the regulation of tight-junction (TJ) assembly [41]. Moreover there are additional targets which are reported to be regulated by HPV encoded proteins. It is now recognized that human cells express a unique family of sense and antisense mitochondrial ncRNAs. Sense transcript or SncmtRNA (SncmtRNA-1), is expressed in normal proliferating cells and cancer cells. Antisense transcripts (ASncmtRNA-1 and -2) are down-regulated which highlights an important step during neoplastic transformation and progression. Details of mechanism involved reveal that immortalization of HFK with HPV-16 or 18 results in repression of antisense transcript through E2 and stimulation of expression of sense transcript via E6 and E7 [42]. E6 and E7 have also been noted to reduce the expression of the globular heads of the C1q receptor (gC1qR), a mitochondrial surface protein [43]. HPV16 E6/E7 are involved in degradation of p130. Recent studies identified that p130 and the related p107 protein are components of a transcriptionally repressive complex termed DREAM (or LINC). In this complex, p130 or p107 are associated with E2F4 or E2F5 and bind to the promoters of genes thus maintaining cell cycle arrest. Sequestration of p130/p107 and E2F4/5 from this complex results is reconstitution of core DREAM proteins via formation of a substitute complex with the B-myb transcription factor that regulates transcription of gene subsets essential for mitosis. Targeted inhibition of HPV16 E6/E7 results in cell-cycle arrest and reformation of the p130–DREAM complex [44].

### Signaling cascades in HPV infected cervical cancer cells

A growing appreciation of misrepresented signaling pathways prompts the realization that spatio-temporal deregulation is likely to contribute broadly to cervical cancer development and may affect the sensitivity and resistance of cancer to targeted therapies. Tremendous experimental work has been done in improving our knowledge that cervical cancer arises from abnormal decision making by cancer cells. These decisions related to cell death or survival are made by molecular signaling networks that process information from outside and from within the HPV infected cervical cancer cells and initiate responses that determine the cell's survival. We dissect this segment of discussion into subheadings that describe regulation of linear signaling cascades in HPV infected cells.

### TGF signaling

**Figure 2:**
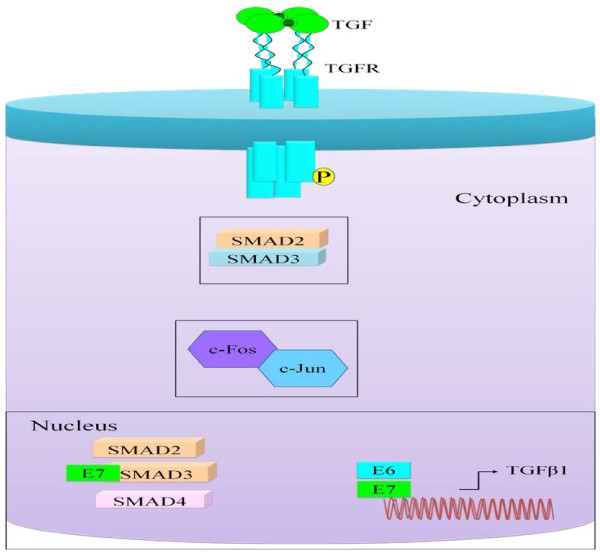
**Shows that HPV infected cervical cancer cells have overexpressed SMAD2/SMAD3 and increased heterodimerization of c-fos and c-jun.** E7 is involved in regulating the expression of TGFβ1. E7 was also suggested to facilitate nuclear localization of SMAD proteins.

Several hints have emerged that indicate that cervical cancer is associated with loss of TGF-β responsiveness (gradual decline in TGF receptors) and because cervical epithelial differentiation is altered by E7. For a better understanding of the underlying mechanisms, status of TGF-β2 and TGF-βRII expression was examined in transgenic mice expressing the oncogene E7 of HPV16 under control of the human Keratin-14 promoter (K14-E7 transgenic mice). The results indicated that there was an overexpression of TGF-β2 and decrease of TGF-βRII expression in this particular model of cervical carcinogenesis [45]. HPV mediates TGFα induced c-fos/c-jun heterodimer formation to regulate expression of oncogenes Figure 2[46]. Surprisingly, there is a research work that illustrates that E6 and E7 encoded by HPV-16 induce activation of TGF beta1 promoter [47]. It was additionally indicated by a contemporary study that inhibition of E7 expression lowered the expression level of TGF-beta1 and induced apoptosis [48]. Detailed structural insights identified that a 9-bp sequence, GGGGCGGGG, representing the consensus Sp1-binding site between -109 and -100 of the TGF-beta 1 promoter, was the major target for E6-mediated transactivation [49]. There is progressive loss of HPV-16 E2 which is higher in CIN3 than in CIN1 or CIN2, and there is a correlation between loss of HPV-16 E2 expression and loss of TGF-beta1 at the lesion site [50]. TGF-beta1 signaling cascade is involved in induction of chromosomal instability in HPV positive cervical cancer cells and inhibition of TGF-beta1 signaling by an inhibitor of TGFRI prevented telomere-mediated chromosomal instability [51].

Overexpression of SMAD2/3 may be involved in the genesis of cervical cancer Figure 2[52]. However this report is in contradiction to another finding that suggested that weak cytoplasmic SMAD4 staining and the absence of Smad4 nuclear staining was associated with poor survival in cervical cancer patients [53]. E7 facilitated the nuclear translocation of Smad proteins (SMAD 2, SMAD3 and SMAD4) in a ligand-independent manner. More intriguingly, E7 interacted with MH1 Domain of SMAD3 to repress TGF-β-mediated transcription Figure 2[27,28]. It is necessary to have a better knowledge of regulation of SMAD subsets by HPV encoded proteins in cervical cancer cells. How these SMAD proteins are degraded or rescued in HPV infected cancer cells to regulate cancer progression still is incompletely understood. SMAD7 heterozygous, silent G to C variant in codon 391 was reported in HPV positive and negative cervical cancer samples. However this report did not identify a relationship between SMAD7 mutation and carcinogenesis [54].

However there is a direct piece of evidence that indicates that TGF-beta1 and IL-4 repress HPV-16 oncogene transcription [55]. Enforced expression of nuclear factor I in TGF-beta-sensitive HPV16-immortalized human keratinocytes (HKc/HPV16) inhibited TGF-beta mediated repression of E6 and E7 [56]. Experiments on transgenic mice provided evidence that HPV-11 transformed xenografts showed up-regulation of TGF beta 1 expression and down-regulation of the expression levels of bcl-2, c-myc, c-Ha-ras, c-jun and NFkB [57,58]. It is noteworthy that HPV-16 transformed cells show down-regulation of bcl-2 and NFkB as well as NFkB function upon TGF beta 1 treatment [57,58].

It still is confusing whether TGF signaling initially acts as a barrier to HPV encoded proteins associated activities. Putting pieces of evidence together indicate contradictory roles of TGF signaling. It appears that TGF signaling is induced in HPV infected cervical cancer cells however other research findings reveal that HPV encoded proteins degrade SMAD proteins to repress TGF signaling. Cervical carcinogenesis was noted in HPV infected cells both in absence and presence of TGF signaling. In depth studies are required to provide a detailed mechanism.

### Wnt signaling

Interestingly, high-throughput technologies, including the analyses of protein networks have considerably enhanced our current understanding that binding of WNTs to frizzled (FZD) and LRP5 or LRP6 co-receptors transduces a signal across the plasma membrane that results in the activation of the Dishevelled (DVL) protein. Activated DVL inhibited the destruction complex and facilitated accumulation of CTNNB1 in the nucleus where it acted as a co-activator for Wnt target genes. Results obtained through immunohistochemistry revealed that normal cervical epithelium showed staining of β-catenin only on the membrane. However, cytoplasmic and nuclear staining was observed only on the basal proliferating layer of the normal stratified squamous epithelium. There is further elaboration of Wnt signaling mediated biological implications and it is clear that activation and stabilization of the catenin is controlled by HPV encoded proteins and various other oncogenes. SV40 small t antigen (smt) was reported to stabilize catenin by inhibiting PP2A [59]. Moreover it is also suggested that HPV encoded proteins stabilize catenin by suppressing SIAH-1. SIAH-1 is a target gene of p53 which is degraded by HPV encoded proteins and targeted inhibition of HPV encoded proteins resulted in restoration of SIAH. Genetic and biochemical data have demonstrated that E6 and E7 facilitated beta-catenin nuclear accumulation [60].

These finding indicated that there is an activated Wnt/β-catenin signaling cascade in HPV-associated premalignant lesions that plays an efficient role in accelerating cervical carcinogenesis. Activation of the Wnt pathway acted as secondary events that are necessary for malignant transformation of HPV-infected epithelial cells [61]. It is also relevant to mention that negative regulators of Wnt signaling are epigenetically repressed and a recent report clarifies an association between DKK3 and SFRP2 promoter methylation in cervical cancer [62].

### Notch Signaling

As discussed in the introductory section that E2 functions as a repressor of the viral upstream regulatory region (URR) promoter that drives transcription of the E6 and E7 oncogenes, therefore loss of E2 is a prerequisite for increased E6/E7 expression. To identify whether the inhibition of E6/E7 expression by activated Notch1 occurs at the level of URR promoter activity, HeLa cells were transiently transfected with a plasmid for the URR promoter and an expression vector for activated Notch1. It was noted that URR promoter activity was significantly reduced by cotransfection of the activated Notch1 expression. Additionally it was observed that Notch-1 repressed URR by inhibiting AP-1. Notch1 inhibited c-Fos protein and simultaneously enhanced another Fos family member, Fra-1. Fra-1 lacked a transcription activating domain and acted as a suppressor rather than an inducer of AP-1-dependent transcription [63].

The data gained through electrophoretic mobility shift assays indicated that Notch overexpression was correlated to altered AP-1 DNA binding activity and complex composition. After inducing a moderate level of Notch expression, an increased DNA binding was demonstrated byAP-1. However cells transfected with high expression levels of Notch displayed a decrease in cFos signal and an increase in Fra1 signal [64]. It is convincing to note that explants of HaCaT cells co-expressing Jagged1and E6/E7 generated tumors greater than 90 mm3. However, co-expression of Delta1 and E6/E7 generated lesions of less than 10 mm3. It was noted that Jagged-1 and E6/E7 co-expressing cancer cells used PI3K/Akt signaling axis to induce EMT [65]. More detailed insights suggest that Jagged-1 induced HES-1 that repressed Manic Fringe (negative regulator of Jagged-1 mediated signaling). These HES1 binding sites were found at nucleotide position −250 upstream of the transcriptional start site of Manic Fringe [66]. Notch-1 is also indicated to behave differently as HPV infected cells use Notch-1 during the progression from cervical intraepithelial lesions (CIN) to invasive cervical carcinoma [67].

### Inducing apoptosis in HPV positive cancer cells

Cellular and molecular studies have outstandingly clarified existing concepts of role of HPV in cervical cancer. It is now evident that HPV oncoproteins transform noncancerous epithelial cells into cancerous carcinomas by targeting key tumor suppressors and pro-apoptotic proteins and additionally impair tumor suppressor and apoptotic pathways. Therefore multi-targeted approach based on targeting of HPV encoded proteins and misrepresented pathways has shown promise in restoring apoptotic pathway. We subdivide next coming section into generalized approaches in inducing apoptosis in HPV infected cervical cancer cells and TRAIL mediated signaling in HPV infected cervical cancer cells. Treating cervical cancer cells with Withaferin A resulted in downregulation of HPV-E6 and E7 oncoproteins [68]. A recent report adds a new dimension to role of HPV-16E6 in cervical cancer cells. It is intriguing to note that enforced expression of 16-E6 in cervical cancer cells stimulated the expression of p53 and induced apoptosis [69]. Interestingly, leaf extract of Bryophyllum pinnata was effective in repressing HPV18 transcription. It also suppressed oncogenic c-Fos and c-Jun expression [70]. n-Hexane and chloroform extracts of Anisomeles malabarica induced death in HPV16-positive cervical cancer cells [71].

### TRAIL mediated apoptosis

Progressively there is a significant accumulation of research reports which have categorized HPV encoded proteins as oncogenes that suppress apoptosis. In the upcoming section we dissect viral encoding genes which have been experimentally investigated regarding their roles in cervical cancer progression and underlying mechanisms which induce resistance against TRAIL mediated apoptosis.

**Figure 3:**
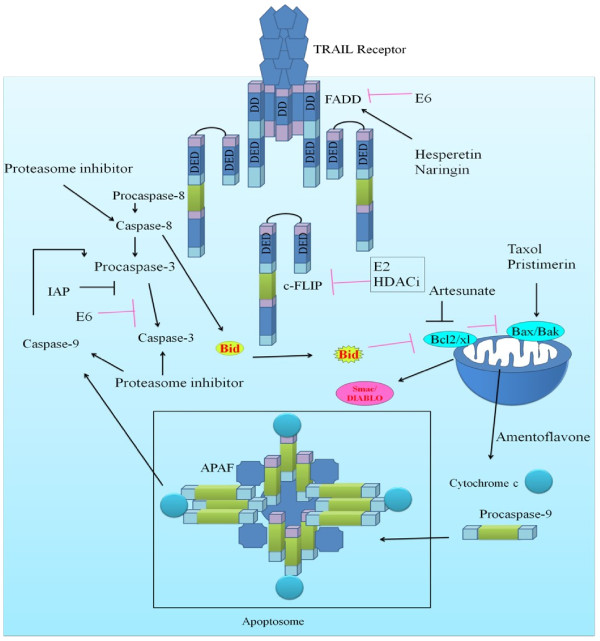
Shows regulation of pro-apoptotic and anti-apoptotic proteins by HPV encoded proteins and compounds.

Cellular studies indicate that TRAIL binds to several distinct receptors and it is a well established piece of information that DR4 and DR5 contain the intracellular death domain (DD) essential for the induction of apoptosis following receptor ligation. Contrary to this, DcR1 nor DcR2 are unable to induce apoptosis due to a complete or partial lack of the intracellular DD, respectively. Using high-throughput technologies, we are able to understand that binding of TRAIL to TRAIL-R1 or TRAIL-R2 induces trimerization of TRAIL-R1 or TRAIL-R2, and FADD binds to the trimerized TRAIL- R1 or TRAIL-R2 death domains. Then, FADD acts as an adaptor molecule that is involved in signal dissemination by recruiting caspase 8, which initiates a proteolytic cascade involving other caspases eventually leading to cell death [72]. TRAIL mediated signaling is shown in Figure 3.

It has lately been shown that pretreating HPV16 E7 expressing cervical cancer cells with HDAC inhibitors considerably sensitized cells to TRAIL. c-FLIP suppression by HDAC inhibitors restores death receptor-mediated apoptosis in HeLa cells. HDAC inhibitors target anti-apoptotic proteins and induce TRAIL mediated apoptosis in resistant cancer cells by enhancing surface expression of TRAIL receptors and re-distribution of TRAIL receptors into lipid rafts (reviewed by [73]). It has previously been shown that E7 oncoprotein binds to multiple functional partners, particularly pRB and HDAC1 and HDAC2 [74]. However, targeted inhibition of HPV16- E7 abolished HDAC inhibitors mediated sensitization to TRAIL [75]. There is a contradictory report that indicates that E6/E7-siRNA induces senescence rather than apoptosis in SiHa cells [76]. Increasing technologies have revolutionized our understanding of the underlying mechanisms which are opted by HPV for the development of cervical cancer, implying that HPVs have evolved immunoevasive mechanisms. It is now known that HPV escapes immunosurveillance by repressing the genes involved in IFN signaling (STAT1), proapoptotic genes (TRAIL), and pathogen recognition receptors (TLR3, RIG-I, and MDA5) [77]. Cells treated with cAMP analog 8-CPT-cAMP (adenylyl cyclase activator), PDE inhibitors (isobutylmethylxanthine) or PKA inhibitors displayed an upregulated expression of Smac/DIABLO. This observation signifies the fact that cAMP/PKA/CREB pathway is an important regulator of Smac/DIABLO transcription [78]. Although it has been shown that HPV encoded E5 protein utilizes cAMP/PKA/CREB pathway to stimulate the expression of genes [31]. It needs to be tested with reference to pro-apoptotic and antiapoptotic gene subsets in cervical cancer cells. E2F1has also been shown to directly bind and activate the promoter of Smac/DIABLO, through the E2F1-binding sites [79].

It is surprising to note that HPV-E2 gene disruption is one of the key features of HPV-induced cervical malignant transformation and is tumor suppressing gene encoded by HPV. Laboratory investigations have revealed that HPV16-E2 inhibits c-FLIP and renders cell hypersensitive to apoptotic signal. It was confirmed by overexpressing cFLIP in cancer cells that completely hampered E2 mediated apoptotic response [80]–[83]. Co-immunoprecipitation and western blot analyses suggest an interaction between HPV 16-E2 and cFLIP isoforms thus inhibiting the recruitment of cFLIP to DISC. Characteristically it has been suggested that targeting of p53 by HPV encoded proteins resulted in transcriptional repression of Puma and abrogation of translocation of Bax to mitochondrial membrane. Puma is a proapoptotic protein that acts as an upstream activator of Bax, by inducing a conformational change thus facilitating the transmigration of Bax from the cytosol to the mitochondrial membrane [84]. Cervical cancer cells treated with cyano analogue of boswellic acid displayed reduced viral E6 mRNA expression and enhanced expression of Puma through p53 pathway [85]. Antisense and peptide aptamers targeting HPV E6/E7 have been shown to induce target cell apoptosis through activation of pRb [86,87]. It has also been shown that p53 triggers the expression of pro-apoptotic proteins (Noxa, Puma and Bax) and repressed the expression of pro-proliferative factors CyclinB1, cdc2, and Cdc25c [88]. Moreover there is study that challenges classical concept of pRb in suppressing cancer via negative regulation of E2F1. It highlights tumor suppressor role of E2F1. E2F1 up-regulates the expression of the pro-apoptotic proteins PUMA, Noxa and Bim. It needs detailed investigation in cervical cancer cells to have a better understanding of the role of E2F1 in cervical cancer progression. Keeping in view tumor suppressor role of E2F1 it will be necessary to identify relationship between pRb, E2F1 and regulation of pro-apoptotic genes [89].

Targeted inhibition of HPV16-E6 resulted in restoration of sensitivity to TRAIL [90]. There is sufficient experimental evidence that transfection of HPV16-E6 gene into cells with wild-type p53, substantially decreased the level of p53 protein, that resulted in suppression of DR4 induction by DNA-damaging agents [91]. Transiently transfecting HPV16-E5 gene into immortalized human keratinocyte cell line HaCaT severely repressed activation of caspase-3 upon TRAIL and FasL treatment [92]. Confluence of information suggests that HPV degrades p53 that results in suppression of p53 mediated expression of death receptors. However there is a finding that shows that IFN-beta increases TRAIL expression both directly at the mRNA level and indirectly by enhancing surface protein levels [93]. HPV16-E6 positive cervical cancer cells displayed a rapid reduction in the protein levels of both FADD and procaspase 8, which resulted in suppression of the activation of caspases 8, 3 and 2 [94]. FZD8 was found to be highly expressed in HeLa cells and in future it would be interesting to note if targeting of FZD8 in cervical cancer cells could be helpful in overcoming resistance against TRAIL [95]. Similar approach has been tested in breast cancer cells and has been shown to be effective [96].

### Multi-targeted approach in restoring TRAIL mediated apoptosis

Researchers are targeting the anti-apoptotic machinery and associated signaling cascades that impair TRAIL mediated apoptosis. Wogonin, a flavonoid isolated from the root of the medicinal herb Scutellaria baicalensis Georgi was reported to be useful in sensitizing cervical cancer cells to TRAIL [97]. It has lately been suggested that bortezomib and nelfinavir considerably enhanced the efficacy of an apoptosis-inducing TRAIL receptor antibody [98]. Aspirin and TRAIL significantly repressed ERK1/2 activation and down-regulated Mcl-1 [99]. Various reports suggest that phosphorylated ERK1/2 induces TRAIL resistant phenotype and aspirin has been shown to inhibit pERK1/2. ERK pathway activation increases the expression of prosurvival proteins, particularly Mcl-1, by stimulating de novo gene expression. It is intriguing to note that expression of Mcl-1 in tumor cells can be regulated at the transcriptional level or through post translational modifications by ERK [100]. Artesunate is an anti-malarial drug that is explored to be effective in sensitizing cervical cancer cells to TRAIL mediated apoptosis by suppressing pro-survival proteins, such as survivin, XIAP and Bcl-XL [101]. Noatbly strong synergistic apoptosis-inducing effect of the combination of rhTRAIL and MG132, particularly in CIN II/III lesions indicates that rhTRAIL combined with proteasome inhibitors open new horizons of therapeutic strategies for CIN II/III [102]. Luteolin synergistically acts with rh TRAIL to induce apoptosis in HeLa cells [103]. HPV control of TRAIL mediated signaling is shown in Figure 3.

### Stimulating the expression of DRs

Phenylethyl isothiocyanate (PEITC) increased the expression of the DR4 and DR5 in cervical cancer cells [104]. Likewise, synergistic treatment with taxol and pristimerin induced cervical cancer apoptosis by enhancing intracellular ROS, upregulation of DR5 and activation of Bax [105]. Cisplatin also enhanced DR5 expression in cervical cancer cells [106]. Irradiation cells showed a p53-dependent rise in DR5 membrane expression [107]. It is surprising to note that proteasome inhibitor MG132 substantially stimulated DR4 and DR5 membrane expression in HeLa. However in SiHa only DR5 membrane expression was upregulated from almost unnoticeable to notable levels independent of p53 [108]. This finding adds a new layer of information that p53 is not indispensible for expression of DR5.

DR5 promoter contains multiple Sp1 binding sites, which may contribute to the increased DR5 expression [109,110]. Sp1 binding sites are also present in promoter region of TRAIL gene [111]. It has also been shown that Sp1 is phosphorylated by ERK that enhanced DNA binding affinity of SP1 [112]. DNMT-mediated hypermethylation of promoter regions cause transcriptional repression and it has been shown that epigenetic repression is induced by DNMT in the proximity of the TRAIL promoter [113]. Moreover, H3K27me3 epigenetic mark at the DR5 promoter represses its expression. However it has been indicated that interference strategies directed against Suz12 and Ezh2 promoted DR5 expression [114]. It is also important to mention that in HPV16 E6 and E7 expressing cervical cancer cells have considerably enhanced DNMT activity and there is a transcriptional down-regulation of E-Cadherin in these cells [115,116]. It has been shown that JNK is involved in stimulating the expression of DR through CHOP and SP1. Using different kinase inhibitors, including the p42/44 MAPK inhibitor PD098059, the p38 MAPK inhibitor SB203580, and the JNK1/2 inhibitor SP600125 it was confirmed that DR5 expression was regulated by JNK. Among the inhibitors tested, the JNK1/2 inhibitor SP600125 effectively impaired DCA-induced DR5 expression, whereas the p42/44 and p38 MAPK inhibitors failed to repress DR5 expression. Cardamonin isolated from black cardamom induces the expression of DRs using CHOP and SP1. The relationship was confirmed by abrogation of CHOP and SP1 that resulted in inhibition of mediated up-regulation of DRs [117]. MEK kinase 1 (MEKK1) is a serine threonine kinase that is activated following etoposide treatment and activates IKK. IKK mediated inactivation of IKB results in sequestration of NF-kappaB from IKB. NFKB translocates into the nucleus to stimulate the expression of DR4 [118]. DR4 is a p53 target gene and is transcriptionally controlled by p53 through a functional intronic p53 binding site (p53BS) [119]. It is also relevant to mention that cells treated with EGF show a decrease in DR5 expression. Detailed analysis indicates that EGF treatment facilitates co-existence of NFKB with HDAC at the binding site present in intronic region of DR5. However etoposide treatment inhibits NFKB mediated recruitment of HDAC to binding site [120]. Cervical cancer cells treated with naringin displayed increased cell surface appearance of DR and mitochondria-mediated apoptosis in human cervical cancer (SiHa) cells Ramesh et al, [121].

It is becoming successively more understandable that nanoparticles (NPs) have become an important tool in many industries including healthcare. Substantial fraction of information has revealed that compared with free antitumor drugs, drug-loaded long-circulating nanovectors show prolonged circulation time in plasma, enhanced accumulation in tumor tissues, and better-quality therapeutic activity. Functionalizing nanovectors with targeting moieties can promote specific receptor-mediated endocytosis, limiting non-specific uptake into the normal tissues. TRAIL has also been conjugated to different nanocarriers to improve the specificity of the delivery system and it has been shown that a nanocomplex system between the positively charged TRAIL and the negatively charged chondroitin sulfate (CS) (CS/TRAIL) was designed and applied in poly (lactide-co-glycolide) (PLGA) microspheres (MSs). The results indicated that TC-loaded PLGA MSs significantly inhibited tumour growth [122]. Furthermore, another recently published work indicated that nanoparticle modified with polyethyleneimine was applied to be a vector of TRAIL for cervical cancer gene therapy [123].

TRAIL resistance has been frequently observed in cancer cells and different approaches are being tested to overcome the TRAIL resistant phenotype. There are different subsets of anti-apoptotic proteins which are over-expressed thus inducing resistance against TRAIL. Results have shown that natural flavonoid chrysin inhibited STAT3 phosphorylation thus repressing transcriptional regulation of Mcl-1. Proof of the concept was provided by treating cervical cancer cells with STAT3-specific inhibitor, cucurbitacin-I, which decreased Mcl-1 levels and enhanced TRAIL-induced cell death [124]. Likewise 5,7-Dihydroxyflavone is a dietary flavonoid has also been reported to overcome resistance against TRAIL by effectively targeting STAT3 phosphorylation. Additionally, Bcl-2, Mcl-1, and IAPs were down-regulated and pro-apoptotic protein Bax was found to be up-regulated [125]. Equol is an isoflavan produced by intestinal bacteria and has been shown to enhance TRAIL-induced apoptosis of HeLa cells through a death receptor-mediated caspase pathway. Data suggested that Equol enhanced TRAIL-induced apoptosis through activation of caspase-3, -8, -9, and cleavage of BID [126].

It is essential to investigate role of HPV encoded proteins in suppressing TRAIL mediated apoptosis. How HPV encoded proteins mediate expression of TRAIL, DR4/DR5 and DcRs is insufficiently studied. It is astonishing to note that HPV16 E2 and E6 are RNA-binding proteins and contain a protein-RNA interaction domain in their C-terminal regions. In addition, E2 and E6 interact with multiple cellular splicing factors like serine/arginine (SR) proteins [127]. This relationship of HPV encoded proteins with regulators of mRNA splicing needs detailed investigation with reference to TRAIL, DRs and subsets of tumor suppressors. Moreover, impairment of TRAIL mediated apoptosis in HPV infected cancer cells needs additional laboratory based experimentations addressing modes of repression of TRAIL and DR4/DR5 at transcriptional and post-transcriptional level. Do HPV encoded proteins recruit silencing machinery at TRAIL and DR4/5 promoters or is there a miRNA mediated regulation of TRAIL and DR4/DR5 or is there an enhanced degradation of DRs are some questions which demand extensive research. Although some cell type specific studies have revealed that c-Cbl-mediated ubiquitination of TRAIL receptors has a main role in the endosomal sorting leading to the degradative pathway. However none of the studies indicated any relationship between HPV encoded proteins in directing degradation of DRs in cervical cancer cells [128]. However it is clear that HPV encoded proteins use ubiquitin ligases (cullin 1 ubiquitin ligase complex) to degrade tumor suppressors [129]. Membrane-associated RING-CH (MARCH) ubiquitin ligase is also reported to ubiquitinate TRAIL-R1 and impairing its cell surface expression [130].

### miRNA and HPV

Integrative genomics and genetics approaches have proven to be a functional tool in elucidating the complex relationships often found in gene regulatory networks and reconstitution of tumor-suppressive miRNA, or sequence-specific knockdown of oncogenic miRNAs by ‘antagomirs,’ has produced favorable antitumor outcomes in experimental models. We discuss existing knowledge gaps that need to be bridged prior to the consideration of miRNA-based experimental cancer gene therapy. These include our incomplete understanding of rate-limiting cellular components that impact the efficiency of this posttranscriptional gene-silencing phenomenon in HPV expressing cervical cancer cells. We partition this section into regulation of miRNAs by p53 and miRNA subsets which are documented to suppress and promote cervical cancer. We partition this section into regulation of miRNAs by p53 and miRNA subsets which are documented to suppress and promote cervical cancer.

### p53 mediated regulation of miRNA subsets in HPV infected cervical cancer

**Figure 4:**
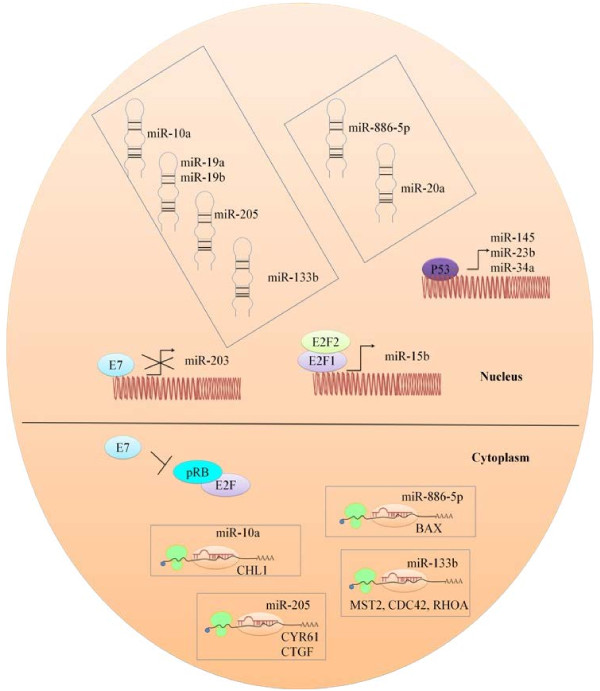
**Shows E7 mediated inhibition of pRB that results in sequestration of E2F.** E2F moves into the nucleus to stimulate the expression of miR-15b. E7 also directly represses miR-203 expression. Additionally, p53 triggers expression of miR-145, miR-23b and miR-34a. Therefore E7 indirectly repress the expression of target miRNAs by degrading p53. miRNAs in slanting boxes are over-expressed in cervical cancer cells. Rectangular Boxes indicate post-transcriptional processing of gene subsets bi miRNAs.

It is now clear that HPV encoded proteins target p53 to inhibit apoptosis of host cells. In the next section we discuss subsets of miRNA which are known targets of p53 and are inhibited by degrading p53. Detailed studies suggested that cortisol induced HPV-E6 expression and suppressed p53 and miR-145 in cervical cancer cells. MiR-145 expression in cervical cancer cells was wild-type p53-dependent, and cortisol down-regulated miR-145 expression [131]. miR-23b and miR-34a were also known targets of P53 however HPV encoded proteins repressed the expression of miR-23b by degrading p53 [80]–[83,132]. Figure 4. miR-15a/miR-16/miR195/miR-497 family, miR-143/miR-145 and the miR-106-363 cluster appeared to be misrepresented in HPV positive cervical cancer cells [133]. HPV encoded proteins regulate expression of miRNAs in infected cells and Figure 4 illustrates the mechanisms.

### HPV encoded proteins use epigenetic machinery writers of sleeping beauty tale of miRNA

HPV encoded proteins use methylation machinery to suppress tumor suppressor miRNAs and there is a direct piece of evidence that reveals hypermethylation of miR-124a and miR-203 in the precursor lesions [134]. There is also considerable evidence regarding increased methylation levels of hsa-miR-124-1 and hsa-miR-124-2 that strongly correlated with reduced hsa-miR-124 expression in cervical tissue specimens [135]. miR-218 was also found to be downregulated [136]. It appears that tumor suppressor miRNA subsets are repressed by installing co-repressor machinery at the promoter regions.

### Tumor suppressor miRNAs

Phosphoinositide 3-kinase catalytic subunit delta (PIK3CD) is a miR-125b target and cells reconstituted with miR-125b represented inhibition of PI3K/Akt/mTOR pathway , while Bid was up-regulated in miR-125b-overexpressing cells [137]. MiR-384-5p is also a known regulator of PIK3CD [138]. MiR-7 has been shown to disrupt PI3K/Akt/mTOR signaling axis [139]. However precise role of miR-384-5p and miR-7 needs to be determined in HPV expressing cervical cancer cells.

miR-17-5p and miR-143 act as tumor suppressors in cancer cells by targeting TP53INP1 and Bcl-2 respectively [140]–[142]. Fascinatingly, overexpression of miR-424 repressed the expression of checkpoint kinase 1 (Chk1) and substantially inhibited cancer progression [143,144]. miR-214 negatively regulates N-acetylgalactosaminyltransferase 7 (GALNT7) and distinctly inhibits cervical cancer cell proliferation, migration, and invasion [145]. miR-372 and miR-223 are down-regulated in cervical cancer and restoration of these miRNAs inhibited cell migration and invasion [146,147]. miR-375 is a tumor suppressor gene and is downregulated in cervical cancer cells however it has been reported that HPV16 E6/E7 does not directly regulate miR-375 expression [80]–[83,148].

**Figure 5:**
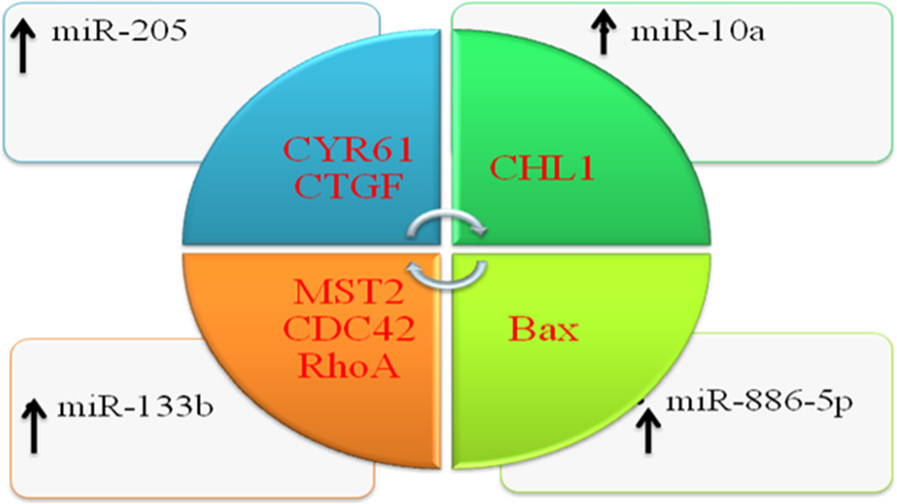
Oncogenic miRNAs which are over-expressed in cervical cancer cells.

It is noteworthy that transiently transfecting pre-miR-34c-3p, in HPV positive cervical cancer cells caused S-phase arrest and apoptosis [149]. It is worth describing that introduction of expression vectors for miR-203 into HPV positive cells substantially limited HPV amplification. It has also been noted that miR-203 expression is regulated through MAPK/PKC pathway and interestingly, this pathway is hampered in E7 expressing cells. Pharmacological activation of PKC pathway is speculated to trigger the expression of miR-203 via AP-1, AP-2, and Sp-1 transcription factor families whose binding sites are present in miR-203. Therefore E7 expressing cells treated with PKC activators did not display an increase in expression of miR-203 [150]. E5 expressing cervical cancer cells showed upregulated miR-146a and repressed miR-324-5p [151]. MiR-497 is a tumor suppressor and targets IGF-1R however it is downregulated in cervical cancer cells [152]. It has been shown that cervical cancer cells treated with mTOR inhibitors displayed an increase in expression of miR-143. It was noted that mTOR was involved in repressing the expression of miR-143 [153]. Additional studies are required to dissect the precise pathway downstream to mTOR that represses the expression. Tumor suppressor miRNA subsets are shown in Figure 5.

### Oncomirs

**Figure 6:**
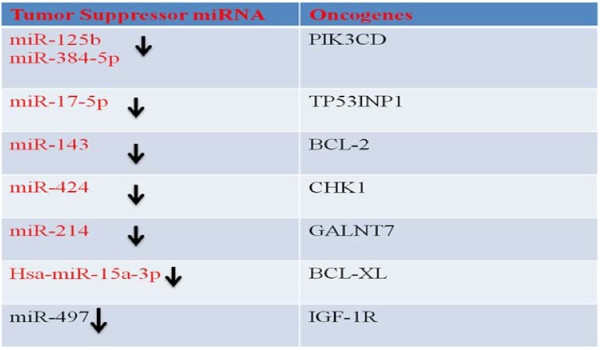
Tumor suppressor miRNAs which are under-expressed in cervical cancer cells.

miR-10a, miR-205 and miR-133b are upregulated in cervical cancer and promote migration and invasion [154]–[156]. CYR61 and CTGF are members of the cysteine rich 61/connective tissue growth factor/nephroblastoma (CCN) family of growth regulators and have tumor suppressing properties. However targeting of CYR61 and CTGF by miR-205 promotes cellular proliferation [155]. CHL1 gene – close homolog of L1, also known as CALL - cell adhesion L1-like encodes a one-pass trans-membrane cell adhesion molecule (CAM) capable of both homotypic and heterotypic binding and has tumor suppressing properties. It is negatively controlled by miR-10a [154]. miR-133b enhances cell proliferation via negative regulation of mammalian sterile 20-like kinase 2 (MST2), cell division control protein 42 homolog (CDC42) and ras homolog gene family member A (RHOA). Additionally, miR-133b overexpressing cells have activated AKT1 and ERK1/2 [156]. Up-regulation of miR-19a and miR-19b promoted cell growth and invasion. The Cullin family member of RING E3 ubiquitin ligases (CUL5) is negatively regulated. Cullin-RING E3 ubiquitin ligase are involved in chaperone-mediated protein regulation and act as tumor suppressors. [143,144]. Therefore it is notable that HPV encoded proteins use various strategies to inhibit Cullin 5 mediated degradation of oncoproteins. miR-20a promoted migration and invasion of cervical cancer cells [157]. miR-886-5p is overexpressed in cervical cancer cells and impair apoptosis by negatively regulating Bax [158]. E7 protein of HPV binds to pRB, a negative regulator of E2F that results in sequestration of E2F from pRB. Binding sites for E2F1 and E2F3 have been identified in the promoter of miR-15b and targeted inhibition of HPV16-E7 resulted in downregulation of miR-15b in cancer cells [159] Figure 4. It has lately been shown that HPV16-positive cancer cells have a downregulated miR-218. Detailed analysis showed that HPV16-E6 oncoprotein suppressed the expression of miR-218 and rescued Laminin 5 β3 (LAMB3). LAMB3 is negatively regulated by miR-218 and cells reconstituted with LAMB3 displayed enhanced migratory potential [160]. Likewise, methylation-mediated transcriptional repression of hsa-miR-149, -203 and -375 is noted in cervical cancer [161]. miR-182 is an oncomir and inhibition of miR-182 in HeLa xenograft mouse model, resulted in tumor growth regression. Moreover expression of miRNA subsets in cervical cancer cell lines displayed two up-regulated (miR-182 and -183) and nine down-regulated (miR-211, 145, 223, 150, 142-5p, 328, 195, 199b, 142-3p) miRNAs [162]. hsa-miR-15a-3p induces apoptosis in cancer cells via negative regulation of Bcl-xL [163]. Similarly, cell reconstructed with miR-214 showed increased expression of Bax, caspase-9, caspase-8 and caspase-3. Moreover, it has been persuasively revealed that miR-214 is regulated by DNA methylation and histone deacetylation [164]. NDRG2 distinctively enhanced Bcl-2 expression and increased the Bcl-2/Bax ratio, which decreased sensitivity of Hela cells to drug-induced apoptosis. However cancer cells expressing miR-15b and miR-16 demonstrated a down-regulated Bcl-2. It is still not know how NDRG2 knock down stimulates the expression of miR-15b and miR-16 [141,142]. Moreover a cell-type specific study indicates that NDRG2 is negatively regulated by miR-650 [165]. Oncogenic miRNA subsets are shown in Figure 6.

There is a complicated network by which miRNA subsets are transcriptionally triggered by downstream effectors of various signaling cascades and in turn miRNA subsets regulate modulators of signaling cascades. How HPV encoded proteins reconstitute signaling, transcriptional and epigenetic machinery to regulate tumor suppressor miRNAs and oncomirs still is a mystery.

### Cervical cancer therapy

On a similar note, Arsenic trioxide induced cervical cancer apoptosis by downregulating HPV-E6 and upregulating p53 [166]. There is a progressive increase in improving the RNA interference strategies. In line with this approach, it has recently been explored that chitosan is appropriate as a carrier for delivery of siRNA into cancer and delivery of chitosan/HPV16 E7 siRNA nanoparticles in vivo is an effective therapy for cervical cancer [167]. E6/E7 specific siRNA-induced transcriptional gene silencing has recently been effectivley tested in cervical cancer cells [168]. Chloroform Extract of Rasagenthi Mezhugu, induced DNA damage and apoptosis in cervical cancer cells [169]. More interestingly, anti-DR5 monoclonal antibody, MD5-1 with a DNA vaccine encoding calreticulin (CRT) linked to human papillomavirus type 16 (HPV-16) E7 antigen (CRT/E7(detox)) provided unique opportunities for the development of therapeutic strategies. The study revealed biological functionality and highlighted that administration of CRT/E7(detox) in mice bearing the E7-expressing tumor, generated the most potent therapeutic anti-tumor effects as well as highest levels of E7-specific CD8+ T cells [170]. There is a finding that has demonstrated a correlation between the shrinkage of HPV16 E6 and E7+ tumors versus DC and LC infiltration in a murine model of cervical cancer thus adding new evidence of the preclinical efficacy of Dendritic cells (DCs) and Langerhans cells (LCs) mediated killing. There is also sufficient evidence that suggests that expression of TRAIL decoy receptors is reduced following introduction of E6 and E7 into host cells [171]. Using different in-vitro strategies, E6 and E7 proteins are targeted to suppress carcinogenesis. These targeted approaches included treatment of cervical cancer cells with biflavonoid amentoflavone, curcumin and Ruthenium oligonucleotides. Cervical cancer cells treated with hesperetin (flavonoid from citrus fruits) displayed an upregulated Fas death receptor and its adaptor protein FADD. Additionally, there was an increased expression of different caspases, p53 and Bax Alshatwi et al, [172].

It was shown that targeted inhibition of E6 and E7 resulted in rescue of p53 Lee et al. [173], Maher et al. [174], Reschner et al. [175]. Moreover, delivery of monoclonal antibodies against E6 in transformed cervical keratinocytes has also been tested. There was an enhanced p53 activity after targeting of E6 Togtema et al. [176]. It needs to be pursued with reference to miRNA subsets which are influenced after treatment with antibodies against E6. Future studies must converge on additional natural compounds with minimal off target effects and considerable efficacy.

GRIM-19 has been acclaimed as tumor suppressor as cells reconstituted with GRIM-19 displayed ubiquitination and degradation of E6AP, and disrupted the E6/E6AP complex. The abrogation of E6/E6AP complex protected p53 from degradation and promoted cell apoptosis [177]. It is impelling to note that phenomenal strides have been made in identifying regulators of cervical cancer. A better understanding of positive and negative regulators will enable the scientists to effectively target oncogenes that promote HPV expression. In line with this approach, it has recently been identified that interaction of mixed lineage leukemia 5 gene (MLL5β) with the AP-1-binding site at the distal region of the HPV18 long control region led to activation of E6/E7 transcription. Targeted inhibition of MLL5β drastically repressed both E6 and E7 expression [178].

In line with this approach, it has been proved that HPV E2 is negative transcriptional modulator of HPV E6 and E7 oncogenes, and also an apoptosis-inducing agent. There is an increasing trend of transiently transfecting tumor suppressor genes into cancer cells to enhance the efficacy of chemotherapy and radiations. A recent report indicated that oncolytic adenovirus armed with human papillomavirus E2 gene in combination with radiation demonstrated considerably augmented antitumor efficacy [80]–[83]. Similarly, pretreatment with dihydrotanshinone increased radiation induced apoptosis in cervical cancer cells through down-regulated HPV E6 gene expression [179]. It has lately been explored that pentoxifylline sensitized human cervical tumor cells to cisplatin-induced apoptosis by inhibiting NF-kappa B and anti-apoptotic proteins [180]. Transgenic mouse model has been developed with malignant cervical lesions allowing the study of the cooperative effect between HPV16-E6/E7 expression and the lack of RXRα in cervical cancer development [181]. This model could be useful to investigate efficacy of chemopreventive and chemotherapeutic strategies. It has been persuasively documented that acetoxychavicol acetate (ACA) with cisplatin (CDDP) worked with effective synergy in HPV-positive human cervical carcinoma cells and induced cell death [182]. HPV encoded proteins control host proteins using an array of post-translational modifications, many of which create binding sites for specific protein-interaction domains thus reconstructing signaling cascades for regulation of cell proliferation. We have discussed common strategies used by HPV encoded proteins for modulation of protein network to impair apoptosis in host cells.

## Conclusion

Signaling networks in cells are composed of upstream and downstream subnetworks. The upstream subnetwork contains the intertwined network of signaling pathways, while the downstream regulatory part controls expression of tumor promoting, tumor suppressing, pro-apoptotic, anti-apoptotic and microRNA subsets in a context dependent manner. HPV encoded proteins have emerged as centrally positioned hubs in regulation of signaling cascades in cervical cancer. Recent studies have revealed an extraordinarily complex network of proteins that is regulated by HPV encoded proteins. This highly interconnected network contrasts our conventional view of the cervical cancer as a linear sequence of events. It has lately been shown that Hh signaling is not induced directly by HPV-encoded proteins instead Hh-activating mutations are selected in cells initially immortalized by HPV [183]. Darinaparsin (ZIO-101, S-dimethylarsino-glutathione) is an organic arsenical and has been noted to effectively inhibit Shh-induced Gli1 expression in cervical cancer cells [184]. It seems that we still lack a broader landscape of linear and integrated signaling pathways in HPV infected cervical cancer cells. SHH signaling related information is insufficient and needs detailed investigation. Coordinate regulation of an miRNA network by a signaling pathway may lead to unpredictable effects on proteins when combinatorial effects are considered, and further exploration of the rules for such interactions are needed in HPV expressing cervical cancer cells. It is appropriate to mention that miRNA subsets under-expressed in cervical cancer cells can be used to regulate the proficiency of cancer-specific adenoviral vector that expressed TRAIL based on miRNA response elements (MREs) of miRNAs whose levels were reduced in cervical cancer. Similar approaches have been tested using in bladder cancer and glioma using adenoviral vector expressing TRAIL and introducing MREs of miRNA subsets down-regulated in respective cancer cells [185,186].

Unquestionably, the growing interest in this class of regulatory RNAs will lead to continued classification of miRNA expression particularly in cervical cancer and recognition of novel miRNA subsets that may act as oncogenes and tumour suppressors. Moreover, it is essential to develop a multi-level cross-talk network of the Notch, Wnt, TGF-β and SHH pathways, identify mutual and pathway-specific components/regulators and predominantly how HPV encoded proteins mastermind cross-talk between these pathways and other pathways.

## Competing interest

All the authors declare that they do not have any conflict of interest.

## Authors’ contribution

TAH and FZ conceived the idea and did literature search on specific points. AAF and TAH did the literature search, integrated different points, and drafted the manuscript. TAH and FZ were involved in discussion and editing the manuscript. AAF designed the diagrams. All authors read and approved the final manuscript.
